# Modeling Neural Adaptation in Auditory Cortex

**DOI:** 10.3389/fncir.2018.00072

**Published:** 2018-09-05

**Authors:** Pawel Kudela, Dana Boatman-Reich, David Beeman, William Stanley Anderson

**Affiliations:** ^1^Department of Neurosurgery, Johns Hopkins School of Medicine, Baltimore, MD, United States; ^2^The Institute for Computational Medicine, Johns Hopkins University, Baltimore, MD, United States; ^3^Department of Neurology, Johns Hopkins School of Medicine, Baltimore, MD, United States; ^4^Department of Otolaryngology, Johns Hopkins School of Medicine, Baltimore, MD, United States; ^5^Department of Electrical, Computer, and Energy Engineering, University of Colorado, Boulder, CO, United States

**Keywords:** computational modeling, auditory cortex, adaptation, neural network, ECoG, repetition suppression, auditory evoked responses, local field potentials

## Abstract

Neural responses recorded from auditory cortex exhibit adaptation, a stimulus-specific decrease that occurs when the same sound is presented repeatedly. Stimulus-specific adaptation is thought to facilitate perception in noisy environments. Although adaptation is assumed to arise independently from cortex, this has been difficult to validate directly *in vivo*. In this study, we used a neural network model of auditory cortex with multicompartmental cell modeling to investigate cortical adaptation. We found that repetitive, non-adapted inputs to layer IV neurons in the model elicited frequency-specific decreases in simulated single neuron, population-level and local field potential (LFP) activity, consistent with stimulus-specific cortical adaptation. Simulated recordings of LFPs, generated solely by excitatory post-synaptic inputs and recorded from layers II/III in the model, showed similar waveform morphologies and stimulus probability effects as auditory evoked responses recorded from human cortex. We tested two proposed mechanisms of cortical adaptation, neural fatigue and neural sharpening, by varying the strength and type of inter- and intra-layer synaptic connections (excitatory, inhibitory). Model simulations showed that synaptic depression modeled in excitatory (AMPA) synapses was sufficient to elicit a reduction in neural firing rate, consistent with neural fatigue. However, introduction of lateral inhibition from local layer II/III interneurons resulted in a reduction in the number of responding neurons, but not their firing rates, consistent with neural sharpening. These modeling results demonstrate that adaptation can arise from multiple neural mechanisms in auditory cortex.

## Introduction

Neural responses decrease when a sensory stimulus is presented repeatedly, a form of short-term neural plasticity known as adaptation or repetition suppression (Li et al., 1993; Desimone, 1996; Grill-Spector et al., 2006). In the auditory system, adaptation that does not generalize to new or rare sounds is termed stimulus-specific (Ulanovsky et al., 2003) and thought to improve auditory perception in noisy environments (Von der Behrens et al., 2009; Taaseh et al., 2011). Although stimulus-specific adaptation occurs at all levels of the auditory system, including the inferior colliculus (Malmierca et al., 2009), it has been studied mainly in cortex. Evidence for adaptation in primary auditory cortex derives from animal studies that show suppression of single-neuron responses to repetitive pure tones (Ulanovsky et al., 2003; Von der Behrens et al., 2009; Farley et al., 2010; Taaseh et al., 2011), but not in simultaneous recordings from the main thalamic inputs to cortex (Ulanovsky et al., 2004; Szymanski et al., 2009). Adaptation has also been observed in human cortical auditory responses recorded from scalp (Briley and Krumbholz, 2013; Lanting et al., 2013) and cortex (Eliades et al., 2014). Although the underlying mechanisms are not fully understood, cortical adaptation is commonly attributed to neural fatigue resulting in decreased neuronal firing rates due to neurotransmitter depletion (Briley and Krumbholz, 2013) or neural sharpening reflecting reduction in the number of responding neurons (Desimone, 1996; Wiggs and Martin, 1998; Henson and Rugg, 2003).

Some studies, however, have reported stimulus-specific adaptation of secondary, or non-lemniscal thalamic inputs to cortex (Anderson et al., 2009; Antunes et al., 2010; Duque et al., 2014). This finding raises the possibility that adaptation is strictly inherited from subcortical sources and does not occur independently in cortex, potentially accounting for the insensitivity of some cortical auditory neurons to repetitive background sounds (noise; Moore et al., 2013; Rabinowitz et al., 2013; Schneider and Woolley, 2013; Mesgarani et al., 2014).

Determining whether adaptation can arise independently from cortical sources is important for elucidating the neural basis of adaptation and for guiding future investigations on the role of adaptation in real-world listening environments where repetitive background sounds (noise) are common. Resolving this issue also has implications for human brain mapping studies that rely increasingly on adaptation paradigms to identify cortical sub-regions of functional specialization (for discussion see Krekelberg et al., 2006; Kar and Krekelberg, 2016). To date, however, it has been difficult to directly test cortical adaptation *in vivo* because cortical input parameters are not readily amenable to experimental manipulation.

In this study, we tested the hypothesis that adaptation can arise independently from cortical sources by using a simple but realistic multi-layer neural network model of auditory cortex that allowed us to systematically control cortical input parameters and circuitry. When repetitive, non-adapted inputs were introduced to multicompartmental layer IV neurons in the model, we observed frequency-specific decreases in simulated single neuron, population-level and local field potential (LFP) activity, consistent with stimulus-specific adaptation. Results were verified by comparison with auditory evoked responses recorded from human cortex under the same experimental conditions. When intra- and inter-layer synaptic connectivity was varied across simulations, we found that synaptic depression modeled in excitatory (AMPA) synapses was sufficient to elicit a reduction in neural firing rates, consistent with neural fatigue. However, introduction of lateral inhibition from local interneurons in layers II/III resulted in a decrease in the overall number of responding neurons but not in their firing rates, consistent with neural sharpening. These results suggest there are multiple, state-dependent mechanisms of adaptation in auditory cortex.

## Materials and Methods

### Neural Network Model

**Figure 1:**
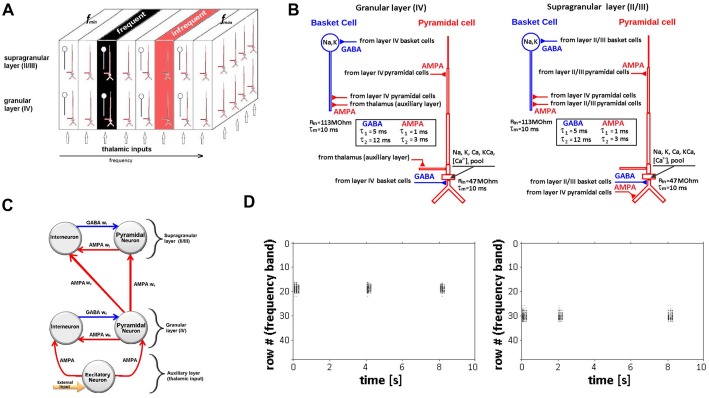
Neural network model of auditory cortex. **(A)** Schematic of multi-layer neural network model design showing the granular cortical layer IV, supragranular layers II/III and an auxiliary layer representing thalamic inputs along with relative locations of frequent and infrequent stimulus. Each layer is represented by arrays of 48 × 48 pyramidal neurons (red) and 24 × 24 interneurons (blue) arranged in 3D space. Frequency-specific (tonotopic) organization is represented by a series of contiguous rows representing specified frequency range (f_min_ − f_max_). **(B)** Simulated neurons in layers IV (left) and II/III (right) are nine-compartment regular-spiking pyramidal cells (red) and two-compartment fast-spiking interneuron (basket) cells (blue). Time-constants for AMPA and GABA synapses are shown in boxes. **(C)** Representation of intra- and inter-layer chemical (AMPA, GABA) synaptic connectivity. **(D)** Raster plot showing row-specific simulated neuronal population activity in response to single, infrequent inputs (rate 2–6 s) to rows 18 (left) and 30 (right).

The network model was designed to represent a continuous 3.6 mm^2^ multilayer patch of primary auditory cortex and was implemented in the GEneral NEural SImulation System (GENESIS 2.4^1^; Bower and Beeman, 1998; Bower et al., 2003). The model has three overlapping layers (arrays) comprising a total of 8,064 simulated neurons: a granular layer IV array, a supragranular layer array representing layers II and III, and an auxiliary layer array at the bottom of the model to simulate auditory afferent inputs from thalamus (Figure 1A). The granular layer array was derived from an earlier single-layer model developed to study cortical waves in primary auditory cortex (Beeman, 2013; Beeman et al., 2017) and expanded to a population of 2,304 excitatory (pyramidal) neurons arranged as a 48 × 48 array with 576 interneurons (24 × 24). The overlapping supragranular array has the same neuronal population composition and configuration. The auxiliary layer array contains 2,304 neurons (48 × 48) representing excitatory thalamocortical afferent inputs to the granular layer. Simulation scripts for the Beeman (2013) single-layer model are available on Model DB accession number 15,067.

The model represents the frequency-specific (tonotopic) organization of auditory cortex, mapping a range of frequencies to the x-coordinates (rows) of each array. Frequencies are mapped from low to high (f_min_ − f_max_) forming contiguous rows, with each row comprising 48 simulated neurons. We use a linear mapping of frequencies to rows as an approximation of the frequency map in a patch of auditory cortex covering a limited frequency range. For the model simulations, the range of frequencies mapped was 800–1,432 Hz (16.63 Hz/row) and included the two tone frequencies (1,000 Hz, 1,200 Hz) used in the experimental recordings. To represent the iso-frequency bands characteristic of primary auditory cortex (Merzenich and Brugge, 1973; Merzenich et al., 1975), the x-coordinate frequency values were held constant across the corresponding y and z coordinates of all three overlapping network arrays (Figure 1A). Because network boundary conditions are not constrained in the model, the five most peripheral rows on each side of the arrays were not mapped to avoid boundary effects. Auxiliary layer neurons were synaptically coupled by their array position to deliver inputs to the immediately overlying network array row to simulate the main pathway of frequency-specific afferent input to auditory cortex that projects from the ventral division of the medial geniculate body of the thalamus. The extent and strength of these connections decay exponentially with the distance for each adjacent row with maximal values in the overlapping row. A single-frequency tone input is simulated in the model as the activation of all neurons positioned in the corresponding (frequency-matched) row of the overlapping auxiliary and granular network arrays. The subsequent spread of the initial activation to adjacent rows of the granular and supragranular arrays is implemented by existing modulatory and feedforward cortical synaptic projections.

#### Model Neurons

Simulated neurons were biophysically realistic multicompartmental cell models. Cell morphology and passive parameters were based on the neocortical pyramidal cell models of Bush and Sejnowski (1993). Regular-spiking pyramidal neurons in layers II/III and IV were modeled as nine-compartment cell models (Figure 1B). Fast-spiking interneurons were modeled as two compartment cells; a single compartment (soma) model was used to represent neurons in the auxiliary layer. Parameters for the regular-spiking and fast-spiking cells were based on prior modeling studies (Traub et al., 1991; Anderson et al., 2007). The modeled regular-spiking neuron included a sodium current *I*_Na_, a delayed rectifier potassium current *I*_K_, a high threshold calcium current *I*_Ca_, a Ca-dependent potassium current *I*_K(Ca)_, and a leakage current *I*_L_. The fast-spiking model neuron had the same set of currents, except for the calcium *I*_Ca_ and Ca-dependent potassium *I*_K(Ca)_ currents. Two types of neuronal activity are simulated in the model: (1) membrane potentials of individual neurons or their aggregates within given rows and layers of the network; and (2) large-scale network activity captured across one entire layer or all layers of the model.

#### Synaptic Connections

Synaptic inputs to excitatory and inhibitory neurons are represented within and between granular and supragranular layers. Within each layer, excitatory neurons are associated with AMPA and inhibitory neurons are associated with ionotropic GABA_A_ chemical synapses (Figure 1C). These connections represent the horizontal (lateral) components of cortical connectivity that function as modulatory feedback pathways targeting apical dendrites of pyramidal neurons positioned in different input rows within a layer (Figure 1B). In granular or supragranular layers, a single-frequency tone will first activate neurons in the corresponding tone-matched row(s) and may then activate, excite or inhibit neurons in adjacent rows as a result of activating their corresponding connections. Post-synaptic currents are described by a double-exponential function with onset times of *t_o_* = 1 ms and 5 ms for excitatory and inhibitory post-synaptic currents respectively (EPSP, IPSP); decay times were *t*_d_ = 3 ms for EPSP and *t*_d_ = 12 ms for IPSP. Synaptic conductance parameter g_syn_ varied from 1 nS to 30 nS for excitatory synapses and from 1 nS to 5 nS for inhibitory synapses. Connection probabilities (*p*) decrease exponentially with radial distance *r* measured between pre- and postsynaptic neurons within a layer as: $p(r)=p_{0}e^{-{\left(r/s\right)}^{2}}$ where *s* = 4 is the scale factor expressed in units of separation between two excitatory cells within a layer. The probability *p*_0_ varies from 0.15 (excitatory-to-excitatory connections) to 0.5 (inhibitory-to-inhibitory connections), as previously described (Levy and Reyes, 2011, 2012; Yuan et al., 2011).

Synaptic connectivity between modeled layers represents the vertical component of cortical connectivity that is intrinsic to cortical minicolumns and serves to simulate afferent feedforward input from granular to supragranular layers (Figure 1C). Pyramidal neurons in the granular layer synapse with interneurons and pyramidal neurons in the supragranular layer. This feedforward input is modeled as inter-laminar synaptic connections targeting basal dendrites of supragranular pyramidal neurons (Figure 1B). The same formula was used to determine the probability of these connections, with *s* = 2 and *p*_0_ varying from 0.5 to 0.8.

The strength of intra- and inter-layer connections is modified dynamically by ongoing network activity according to a phenomenological model of short-term synaptic plasticity (STP; Markram and Tsodyks, 1996; Varela et al., 1997, 1999; Wang et al., 2006). Although the STP model can produce either depression or potentiation of synaptic strength, we focused exclusively on synaptic depression because studies of mouse auditory cortex (Levy and Reyes, 2012) have showed strong short term depression (STD) on AMPA synapses of both pyramidal cells and fast-spiking inhibitory cells. For AMPA synapses in the model, this reported STD was modeled with fast (D_1_ = 0.46; τ_D1_ = 0.38 s and slow (D_2_ = 0.76; τ_D2_ = 9.2 s) synaptic depression factors (Varela et al., 1997). Network neurons were simulated in the absence of background firing activity to facilitate identification and for better visualization of stimulus driven adaptation in neuronal activity (Bernacchia, 2014; Kudela and Anderson, 2015), after confirming that background activity, i.e., 5–8 Hz spontaneous firing rate (Munguia et al., 2013), did not alter the results. Secondary inputs to layers IV and II/III, including other inter-laminar, inter-regional and commissural inputs and background firing were omitted from the model to allow direct examination of the main thalamic inputs to auditory cortex. GENESIS implementation of the Varela et al.’s (1997) STP model are at the repository for the continued development of the GENESIS 2.4 neural simulator^2^.

#### Model Tuning

Single 50-ms pulse inputs to different rows of the network elicited single neuron and population level action potentials that were spatially restricted to the stimulated row (Figure 1D). The “row” specific nature of these responses is consistent with the frequency-specific responses characteristic of neurons in primary auditory cortex and confirms the functional tonotopic organization of the model.

#### Model Simulations

Three batch simulations were performed to investigate effects of stimulus repetition on the network model. All simulations were based on the same experimental adaptation paradigm that was used in the human intracranial recording studies. The experimental paradigm is a 300-trial passive auditory oddball task used to present two 200-ms duration single-frequency (pure) tone stimuli: 1,000 Hz and 1,200 Hz which are readily distinguishable by the human ear. Oddball paradigms are commonly used to study neural adaptation in both human and animal studies (Ulanovsky et al., 2004; Von der Behrens et al., 2009; Eliades et al., 2014; Malinowska et al., 2017). The 1,000 Hz tone was designated as the high-probability (repetitive) stimulus and presented consecutively (2–12 repetitions; 82% of total number of trials); the low-probability 1,200 Hz tone was interspersed infrequently (12% trials) and non-consecutively among the repetitive stimulus trials. The tone stimuli were presented on sequential trials at an inter-stimulus interval of 1,200 ms to match the experimental recording paradigm and for consistency with other human auditory adaptation studies (Lanting et al., 2013; Eliades et al., 2014).

*Simulation 1* was implemented to test whether repetitive, excitatory (e.g., non-adapted) input to layer IV model neurons leads to the reduction (adaptation) of single-neuron and population-level responses. We used the oddball paradigm to deliver a repetitive pulse input at a high-probability rate of 0.5–1 Hz for a period of 10 s, interspersed by a second low probability input of 0.1–0.2 Hz. The high- and low-probability inputs were delivered to simulated neurons centered on two different rows of the layer IV array: rows 18 and 30 approximating pure tone inputs of 1,000 Hz and 1,200 Hz, respectively. AMPA synapses were modeled with fast D_1_ = 0.46 and slow D_2_ = 0.76 synaptic depression factors in the STP model (Varela et al., 1997). Both single neuron and population level (across-row) responses were simulated. To control for potential effects of input location on neuronal responses, akin to intrinsic differences in the frequency preferences of neurons along the tonotopic gradient in auditory cortex, the stimulation was re-run switching the location of the high and low probability input rows.

*Simulation 2* was conducted to simulate repetition effects on LFP recordings for comparison with experimental recordings (see below). Model input parameters were matched to those of the experimental oddball paradigm. A simulated electrode was positioned at height *z* = 1 mm above the bottom of the supragranular layer (II/III) in extracellular space to represent LFP recordings. The simulated electrode was located above the middle of the iso-frequency band (x-y plane) centered at rows 18 and 30. The LFP was calculated as the sum of transmembrane and capacitive currents from all neuronal compartments of all responding neurons in the network using the GENESIS efield object, taking into account the distance between the electrode and a given neuronal compartment (Nunez, 1981; Nunez and Srinivasan, 2006). The medium in which neurons are embedded is treated as homogenous without capacitance effects. LFPs were generated separately for frequent (repetitive) and infrequent inputs and averaged in the time domain for comparison with human auditory evoked responses.

*Simulation 3* was performed to test two candidate mechanisms of adaptation: neural fatigue and neural sharpening. The neural fatigue account predicts a decrease in overall neuronal firing rate due to decreased excitatory synaptic inputs, while neural sharpening predicts a decrease in the number of responding neurons due to changes (increases) in inhibitory synaptic inputs. The main dependent variables were the total number of action potentials fired (firing rate) and the total number of responding neurons. We first ran Simulation 3 with excitatory (AMPA) synaptic inputs and without inhibitory synaptic inputs (weak GABA synaptic weights) e.g., matching all parameters to Simulation 1. We then re-ran Simulation 3 without excitatory synaptic inputs implemented by AMPA modeled as non-depressing synapses. To examine effects of inhibitory synaptic inputs on adaptation, we next ran Simulation 3 with both excitatory and inhibitory synaptic inputs. Inhibitory inputs were implemented via lateral (intra-layer) interneurons.

### Experimental Recordings

Model simulated LFPs were compared with intracranial electrocorticographic (ECoG) recordings from a normal-hearing, right-handed, adult male epilepsy patient undergoing intracranial monitoring for clinical purposes of seizure localization. ECoG signals were recorded from subdural electrodes (2.3 mm diameter, 9 mm spacing) embedded in an 8 × 8 array that was implanted over the lateral cortical surface of the right hemisphere (Figure 3A). The passive auditory oddball paradigm was used to present the tone stimuli at a comfortable listening level through binaural insert earphones while the patient watched an animated movie with no sound. ECoG recording parameters are described in detail elsewhere (Boatman-Reich et al., 2010; Eliades et al., 2014). The patient provided informed consent for the auditory ECoG recordings in compliance with Johns Hopkins Institutional Review Board requirements. The study was approved by the Johns Hopkins Institutional Review Board.

**Figure 2:**
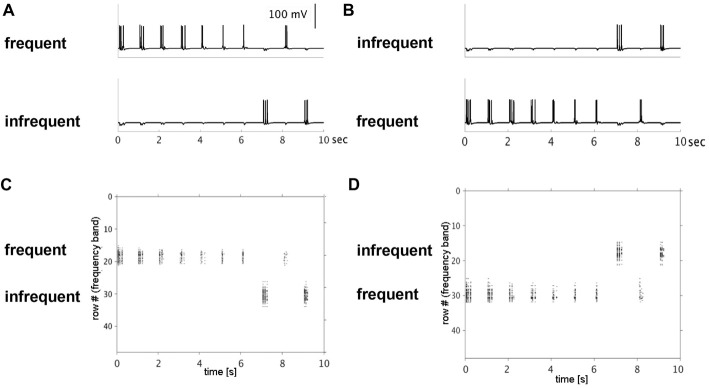
Simulated single neuron and neuronal population action potentials. Top row shows decreased single-neuron firing with repetitive (frequent) presentation of a 50-ms pulse to rows 18 **(A)** or 30 **(B)** over a 10-s period. The decrease in firing rates was row-specific, simulating stimulus-specific adaptation. Note: no decrease in firing rate was observed when the same input pulse was presented infrequently to rows 30 **(A)** or 18 **(B)**. Bottom row shows raster plots of population-level firing activity corresponding to the same row and input conditions (top row). Decreased population-level firing activity (horizontal extent), was observed with repetitive inputs to row 18 **(C)** and row 30 **(D)**.

**Figure 3:**
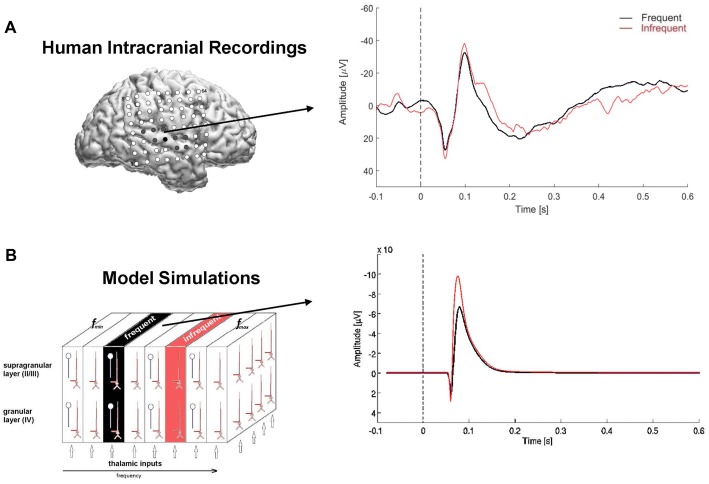
Model simulated local field potentials (LFPs) and human auditory evoked responses. **(A)** Lateral view of the right hemisphere showing 8 × 8 subdural electrode array (top left); electrode positions are co-registered to the individual 3D MRI brain reconstruction. Filled electrodes denote sites that were in auditory-responsive cortex. Arrow points from electrode used to compute the auditory evoked response (right). The auditory evoked response was derived separately for the repetitive (frequent, black) and infrequent (red) stimuli and overlaid for comparison. Time is on the x-axis in seconds, with dashed line at 0-ms denoting stimulus onset; amplitude is on the y-axis with negative deflections pointing upwards e.g., above zero. **(B)** Model simulated LFP using the same input paradigm as the experimental recordings. Note: pulse inputs were delayed by 50 ms to account for neural transmission delay between ear and cortex in the human auditory system.

Auditory evoked responses were computed for each electrode channel by averaging trials in the time domain, based on stimulus probability (frequent, infrequent). Identification of an N1 response in the averaged waveforms was used to confirm the presence of an evoked response. The evoked N1 response is a robust, vertex-negative deflection that peaks around 100 ms after stimulus presentation and is thought to reflect an early, automatic cortical response to sound, with neural generators in primary auditory cortex. For comparison with the model simulated LFPs, we used auditory evoked responses recorded from an electrode located directly over the posterior section of the Sylvian fissure, corresponding to auditory cortex (Figure 3A).

## Results

Model simulation results are presented in this section. In Simulation 1, we investigated effects of repetitive, non-adapted inputs on the model’s neural firing patterns. Simulated membrane potential traces from one pyramidal cell in each of the two input rows and raster plots of the aggregate across-row population activity at the supragranular layer are shown in Figure 2. For the high-probability input stimulus (row 18), we observed a reduction of up to 50% in simulated single neuron and population level firing rates. Conversely, no reduction was observed at the single neuron or population level for the low-probability input stimulus (row 30). When the simulation was re-run switching the high- and low-probability inputs between the two rows, we again observed a decrease in single neuron and population-level firing activity within the high-probability input row (now row 30), but not in the low-probability input row (now row 18). These results indicate that adaptation of simulated single neuron and population level responses occurs independently in cortex and is stimulus-specific.

Simulation 2 was conducted to derive simulated LFPs for comparison with ECoG recordings acquired directly from human cortex under the same experimental conditions. As shown in Figure 3, both simulated and experimental responses to highly repetitive stimulus inputs were reduced compared with responses to low-probability inputs, consistent with stimulus-specific adaptation of the high-probability response. Similarly, both the trial-averaged simulated LFPs and human auditory evoked responses to high- and low-probability stimuli comprised bi-phasic waveforms: positively deflecting peaks followed by negatively deflecting peaks. The peak of the initial positive deflection in the human evoked response waveforms occurred around 55–60 ms post-stimulus, consistent with the evoked P1 response. The subsequent negative deflection, peaking around 100–105 ms post-stimulus, was identified as the N1 response. Neural generators for both the P1 and N1 are located in primary auditory cortex; the P1 also has neural generators in thalamus.

**Figure 4:**
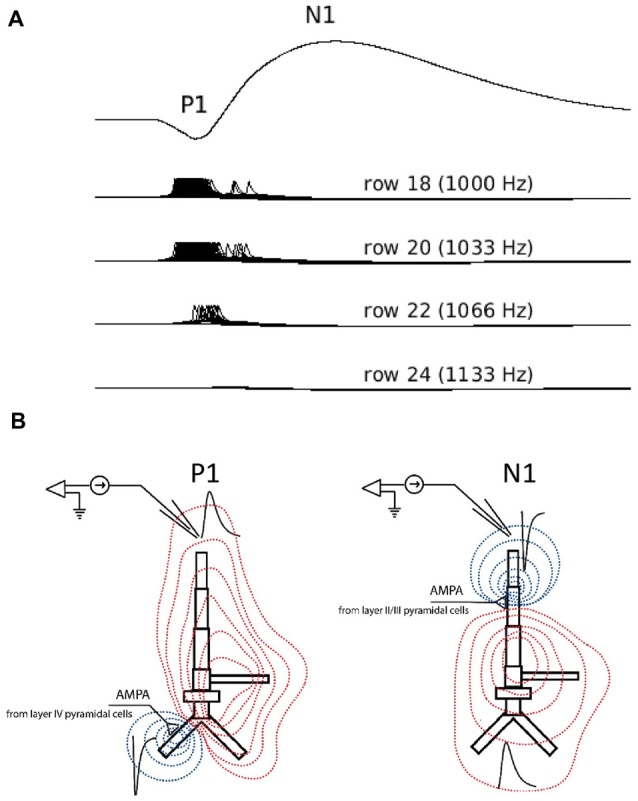
Simulated LFPs showing P1 and N1 generation in the model. **(A)** Simulated averaged evoked responses to frequent inputs plotted along with single-neuron activity of selected neurons in rows 18 (frequent tone), 20, 22 and 24. The smaller amplitude positive deflection (P1) reflects the arrival of excitatory synaptic inputs at basal dendrites of layer II/III pyramidal neurons in row 18 through feedforward connections from layer IV. These inputs cause the initial neuronal firing of neurons in row 18. The subsequent negative deflection (N1) reflects the arrival of all excitatory post-synaptic inputs at the apical dendrites of pyramidal neurons in rows adjacent to row 18 and through lateral feedback connections in layer II/III. These lateral feedback inputs are secondary to neuronal activity in row 18. Note: by convention, negative LFP deflections are shown pointing upward. **(B)** LFP traces (black) in response to excitatory synaptic current inputs (sinks) injected into the basal (left) and apical (right) dendrite of a layer II/III model of pyramidal neuron. Red and blue contour lines correspond to positive and negative values for the LFP amplitude, respectively. The position of electrode corresponds to the position of electrode in Simulation 2.

The corresponding “P1-N1” peaks in the simulated waveforms were generated solely by excitatory postsynaptic activity in supragranular pyramidal neurons (Figure 4). The first simulated positive waveform peak coincided with activation of pyramidal neurons in the supragranular layer by excitatory feedforward projections from the granular layer (Figure 4). The positive voltage deflection reflected the synchronized arrival of EPSPs at basal dendrites of pyramidal neurons in supragranular layers II/III and their vertical propagation towards the cell somas and the simulated electrode positioned above the supragranular layer. The negative peak in the simulated waveform emerged approximately 30–40 ms later and co-occurred with the arrival of EPSPs at the apical dendrites of pyramidal neurons in the supragranular layer. Barrages of EPSPs were generated at apical dendrites by lateral (intra-layer) input, representing modulatory cortical feedback, and were secondary to EPSPs at the basal dendrites. The negative waveform deflection reflected the propagation of EPSPs in the opposite direction along apical dendrites: towards the cell somas and away from the electrode. The broader width of the negative waveform suggests that arrival of EPSPs at apical dendrites of pyramidal neurons may be less temporally correlated than at basal dendrites. Repetition-related decreases in P1 and N1 amplitudes reflect dynamic adjustment (depression) of AMPA synaptic connections resulting in the weakening of synaptic inputs from layer IV to layer II/III pyramidal cells with repetition.

A notable difference between the simulated and human LFPs is the lack of identifiable second positive peak (P2) response in the simulated waveforms. In human recordings, including ours, the P2 is a vertex-positive response that follows the N1 response and occurs around 200 ms post-stimulus with its neural generators located outside of primary auditory cortex in higher auditory areas. We speculate that the absence of a P2 response in the simulated waveform reflects the lack of synaptic inputs to the model from non-primary auditory areas, including the lateral superior temporal gyrus. Similarly, a second negative peak occurring around 150 ms in the human LFP response (N2) to the infrequent tone was not observed in the simulated LFP likely also reflecting the lack of synaptic inputs from non-primary auditory sources in the model.

**Figure 5:**
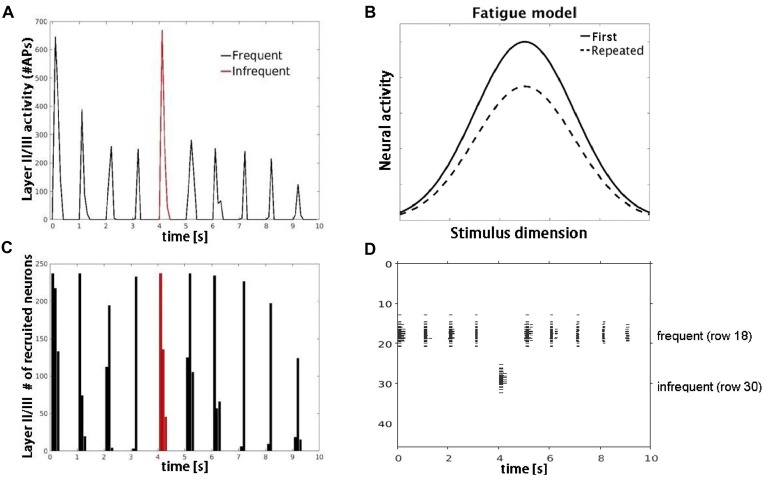
Model simulations with AMPA modeled synaptic depression. **(A)** Plot shows decrease in number of APs fired by layer II/III pyramidal cells with repetitive (black) but not infrequent (red) inputs. **(B)** Schematic of the neural fatigue model adapted from Grill-Spector et al. (2006). Tuning curves of responses before and after stimulus repetition. Repeated stimulus corresponds to center of tuning curves along stimulus dimension axis. **(C)** Plot showing number of neurons responding to the frequent (black) and infrequent (red) stimulus. **(D)** Raster plot of neuronal population activity showing decrease in response firing rate (horizontal extent) but not in the number of neurons firing.

**Figure 6:**
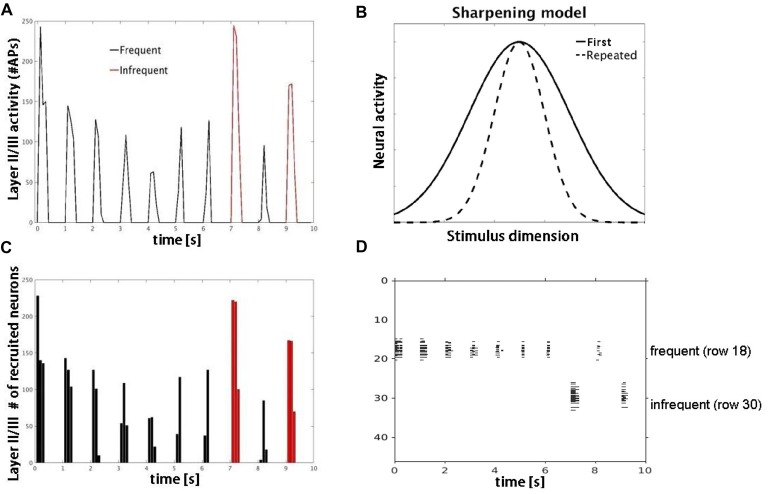
Model simulations with lateral inhibition. **(A)** Plot shows number of APs fired by layer II/III pyramidal cells in response to frequent (black) vs. infrequent (red) inputs. **(B)** Schematic of sharpening model adapted from Grill-Spector et al. (2006). Repeated stimulus corresponds to center of tuning curves along stimulus dimension axis. **(C)** Plot shows decrease in number of responding neurons consistent with neural sharpening account. **(D)** Raster plot of neuronal population activity showing decrease in number of responding neurons (vertical extent) but not firing rate.

Simulation 3 tested two competing accounts of adaptation: neural fatigue and neural sharpening. With repetitive inputs, we observed a decrease in single cell and population level firing rates in or close to the corresponding row (row 18), but not in the row that received infrequent inputs (row 30), as shown in Figure 5. The average decrease in firing rate from the first to the second repetition was 40%. When the simulation was re-run with AMPA modeled as non-depressing synapses but all other parameters the same, we observed no corresponding decrease in firing rates with repetitive inputs (data not shown). These results suggest that firing rate adaptation is associated with synaptic depression modeled in AMPA synapses, consistent with the neural fatigue account. However, when inhibitory inputs were introduced within both granular and supragranular layers, simulated responses to repetitive input (row 18) showed little to no reduction in firing rate, but instead a decrease in the number of responding neurons (Figure 6). No reduction in neural activity in the array row that received the infrequent stimulus input was observed, except for two consecutive stimuli separated by one frequent stimulus. The average decrease in the number of responding neurons from the first to the second repetition was 38%. These results suggest that lateral inhibition from local interneurons results in a sparser neuronal representation with repeated stimulus inputs consistent with the neural sharpening account. The decrease in the number of responding neurons was most evident in layers II/III, in agreement with a prior computational modeling study of adaptation in medial temporal cortex (Norman and O’Reilly, 2003). Taken together, results from Simulation 3 suggest that neural fatigue and sharpening accounts may not be mutually exclusive, but rather co-exist in auditory cortex and are state-dependent i.e., determined by the balance of inhibitory and excitatory connections.

## Discussion

We implemented a simple, but biologically realistic neural network model to investigate adaptation in auditory cortex. Studies of adaptation in previous models were limited almost exclusively to analyses of spike population responses (i.e., mean spike counts). The novelty of our network model lies in its ability to model adaptation at the level of cortical evoked responses, and in addition to population and single-neuron levels. Model simulations showed decreased single-neuron, population-level and LFP activity when repetitive, non-adapted inputs were introduced to layer IV pyramidal neurons. The observed decreases in neuronal activity were row (frequency) specific in that they did not generalize to other rows in the model. These results demonstrate that stimulus-specific adaptation can arise from independent cortical sources. Simulated LFP adaptation profiles were verified by comparison with auditory evoked responses recorded from human cortex and are consistent with prior adaptation studies (Ulanovsky et al., 2004; Farley et al., 2010; Briley and Krumbholz, 2013; Lanting et al., 2013; Eliades et al., 2014; Kar and Krekelberg, 2016). Importantly, although our results support independent cortical mechanisms of adaptation, they do not preclude other potential sources of adaptation, such as secondary (non-lemniscal) inputs from thalamus (Anderson et al., 2009; Antunes et al., 2010). Future modeling studies may be useful for elucidating the relationship between cortical and other sources of adaptation.

An unexpected finding from the LFP simulations was that both bi-phasic (P1-N1) peaks in the simulated waveform were generated from excitatory postsynaptic activity of supragranular pyramidal neurons. This contrasts with previous reports that these bi-phasic deflections reflect sequences of excitatory (P1) followed by inhibitory (N1) post-synaptic inputs (Tan et al., 2004; Oswald et al., 2006). However, a similar finding was reported in a recent study of rat somatosensory cortex where reduced inhibitory post-synaptic activity affected only the late phase of the N1 by increasing its peak and duration (Bruyns-Haylett et al., 2017). Based on the time course and morphology of the simulated LFP components, we speculate that the initial positivity (P1) reflected bottom-up activity of the basal dendrites of pyramidal neurons in layers II/III as shown in Figure 4. The subsequent negative deflection (N1) occurred 30–40 ms later, reflecting the arrival of excitatory post-synaptic inputs at the apical dendrites of layers II/III pyramidal neurons through lateral feedback connections. The excitation that gives rise to the N1 is terminated by subsequent inhibition from interneurons. To our knowledge, this is the first demonstration that the bi-phasic, P1-N1 components of the LFP may reflect differences in the location of excitatory postsynaptic inputs to dendritic targets (basal vs. apical) rather than alternating sequences of excitatory and inhibitory inputs, and will need to be confirmed by future studies.

We next used the model to examine effects of excitatory and inhibitory synaptic inputs on adaptation, measured as changes in neuronal firing rate and number of responding neurons. When synaptic depression was modeled in AMPA synapses, we observed a decrease in single-neuron and population-level firing rates with repetitive stimulus inputs, consistent with effects of neural fatigue. However, when inhibitory inputs were introduced, simulated responses to repetitive inputs showed little to no reduction in firing rate, but instead a decrease in the number of responding neurons, consistent with effects of neural sharpening. These results suggest that lateral inhibition contributes to cortical adaptation not by decreasing neuronal firing rates through modulatory feedback, but rather by reducing the size of the responding neuronal population. The decrease in the number of responding neurons was most evident in layers II/III, in agreement with a prior computational modeling study of visual adaptation and recall in medial temporal cortex (Norman and O’Reilly, 2003).

These modeling results demonstrate that cortical adaptation can arise from either neural fatigue or neural sharpening and that these mechanisms co-exist and are state-dependent i.e., determined by the balance of inhibitory and excitatory connections or by the duration of neural responses. For example, the range of adaptation that initially arises from reduction in firing rate (neural fatigue) could be effectively extended by a reduction in number of responding neurons (neural sharpening) as the neuronal firing pattern becomes sparse. Our results help to reconcile prior competing accounts of adaptation and are consistent with recent ECoG findings showing multiple sources of adaptation in human auditory cortex (Malinowska et al., 2017).

The novel aspects of our study are three-fold: (1) the neural fatigue and sharpening mechanisms are shown to rely exclusively on the firing rate of neurons, a finding that was predicated on using a neuronal spiking model; (2) adaptation behaviors were observed in a network composed of morphologically realistic, multicompartmental neuron models; such models are required in order to generate extracellular field potentials; and (3) simulated cortical field potentials were compared directly to experimental recordings from human cortex. To our knowledge, this has not been done previously. Although other studies have used computational models to investigate adaptation in auditory cortex (Mill et al., 2011; Wang and Knösche, 2013; May et al., 2015; Yarden and Nelken, 2017), most were not designed to be biologically realistic in terms of the morphological detail needed to represent extracellular field potentials recorded *in vivo*. In contrast, our model uses multicompartmental representations of excitatory and inhibitory neurons. This approach is useful for modeling LFPs that are produced by spatially separated currents that enter and leave cells at different points along the dendritic trees. Inclusion of realistic spiking neuron representations also allowed us to implement models of STP that can be fit to measurements *in situ* in auditory or other sensory (e.g., somatosensory) cortices. Other realistic features of the model include the laminar (multi-layer) architecture, intra- and inter-layer synaptic connectivity, and tonotopic organization characteristic of primary auditory cortex. These features were important for investigating the specific neural mechanisms of adaptation.

The current implementation of our model has several limitations that warrant mention. A number of features have been simplified or omitted to reduce the number of model parameters, including gap junction inhibition between basket cells, slow GABAergic, and NMDA receptor-mediated synaptic transmissions. Although NMDA synapses provide slow long-term potentiation (LTP), the timescale of the observed adaptation suggests that short-term depression is more likely the dominant factor (please see Supplementary Figure S1 for further details). Indeed, our initial attempts using a spike-timing-dependent plasticity model for LTP produced effects that were too long lasting. Another potential limitation is that although our model was designed to represent auditory cortex without associated subcortical nuclei, we cannot rule-out potential subcortical contributions at the spiking level. However, adaptation of simulated LFPs in the model was attributed exclusively to cortical sources. Specifically, our model offers an explanation for how different biphasic components of the evoked potentials are generated in upper cortical layers and why these evoked responses decrease with stimulus repetition. Finally, as noted earlier, the model also does not include layers V/VI that provide secondary input to layer IV neurons or secondary cortical and laminar inputs to layers IV and II/III. The model will be expanded to include these features in future implementations.

## Summary

We used a neural network model with multicompartmental cell representation to investigate neural mechanisms of adaptation in auditory cortex. Model simulations demonstrate that adaptation can arise independently from cortical sources and is supported by multiple, state-dependent neural mechanisms. Our findings highlight the utility of using computational modeling to study the neurobiological bases of complex cortical functions such as adaptation.

## Author Contributions

PK, DB and WSA designed and implemented the cortical modeling studies. DB-R performed the experimental recordings and data analysis. PK and DB-R drafted the manuscript. All authors contributed to manuscript revision, read and approved the submitted version.

## Conflict of Interest Statement

The authors declare that the research was conducted in the absence of any commercial or financial relationships that could be construed as a potential conflict of interest.

## Footnotes

^1^http://www.genesis-sim.org/GENESIS/

^2^https://github.com/genesis-sim/genesis-2.4
