# Supplementary material for: Modeling Neural Adaptation in Auditory Cortex
Source: Front Neural Circuits. 2018 Sep 5;12:72. doi: 10.3389/fncir.2018.00072 (PMC6133953; doi:10.3389/fncir.2018.00072)
Supplement: Supplementary file 1 [file Data_Sheet_1.docx]

**
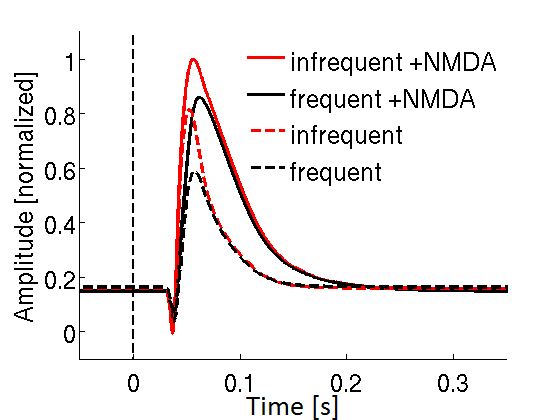
**

**Figure S1.** Comparison of model simulated evoked responses (ERs) derived from local field potentials in model simulations with and without NMDA receptor-mediated synapses. AMPA/NMDA connection ratio for pyramidal to pyramidal neurons was 1, g_NMDA_/g_AMPA_ = 0.1, and rise and decay-times of NMDA currents were 3 and 50 ms respectively. NMDA receptor-mediated synapses deliver more excitation and produce larger amplitude of ERs (20% for infrequent, 35% for frequent) and slower (25%) N1 decay. However, these effects may be less prominent after re-tuning AMPA and GABA conductances in the model. The simple addition of the NMDA induced excitation to the model alters the balance of excitation vs. inhibition in the model. Even though, it is clear that ER amplitude increase for non-adapted stimulus is smaller than ER amplitude increase for adapted stimulus suggesting a dominant role of AMPA and little (constant) contribution of NMDA synapses to adaptation.
